# Electromyographic and Joint Kinematic Patterns in Runner’s Dystonia

**DOI:** 10.3390/toxins10040166

**Published:** 2018-04-20

**Authors:** Omar F. Ahmad, Pritha Ghosh, Christopher Stanley, Barbara Karp, Mark Hallett, Codrin Lungu, Katharine Alter

**Affiliations:** 1 National Institute of Neurological Disorders and Stroke, National Institutes of Health, Bethesda, MD 20892, USA; ahmadof@ninds.nih.gov (O.F.A.); hallettm@ninds.nih.gov (M.H.); lunguci@ninds.nih.gov (C.L.); 2 Department of Neurology, George Washington University Medical Faculty Associates, Washington, DC 20037, USA; pghosh@mfa.gwu.edu; 3 Functional and Applied Biomechanics Laboratory, National Institutes of Health, Bethesda, MD 20892, USA; stanleycj@cc.nih.gov; 4 Combined Neurosciences IRB, National Institutes of Health, Bethesda, MD 20892, USA; karpb@ninds.nih.gov

**Keywords:** runner’s dystonia, task specific dystonia, lower extremity dystonia, kinematics, electromyography, gait analysis, coactivation, compensatory activity, lateral heel whip, botulinum toxin

## Abstract

Runner’s dystonia (RD) is a task-specific focal dystonia of the lower limbs that occurs when running. In this retrospective case series, we present surface electromyography (EMG) and joint kinematic data from thirteen patients with RD who underwent instrumented gait analysis (IGA) at the Functional and Biomechanics Laboratory at the National Institutes of Health. Four cases of RD are described in greater detail to demonstrate the potential utility of EMG with kinematic studies to identify dystonic muscle groups in RD. In these cases, the methodology for muscle selection for botulinum toxin therapy and the therapeutic response is discussed. Lateral heel whip, a proposed novel presentation of lower-limb dystonia, is also described.

## 1. Introduction

Runner’s dystonia (RD) is a task-specific focal dystonia of the lower limbs that occurs when running [1]. Symptoms manifest in adulthood and present in established long-distance runners who have been running for several years [2,3]. Task specificity and sensory tricks may be present initially and may fade over time. Spread to adjacent muscle groups or body regions is common, yet the dystonia rarely generalizes. Patients may note a loss of automaticity, requiring conscious mental effort for every step. They often experience slowed running speeds, and an inability to change speeds or direction. Patients with RD sometimes switch to other sports to maintain their prior level of athletic performance. 

In patients with RD, in order to maintain posture, balance, and forward momentum while running, the “normal” leg must compensate for the dystonic posture in the affected leg [4]. Compensatory activity [5] has been identified in various gait disorders [6,7,8] as well as in other task-specific dystonias involving the upper limbs [9]. Distinguishing dystonia from compensatory activity is critical when developing a treatment strategy with botulinum neurotoxin (BoNT) injections [4,10]. However, judging whether a specific movement is dystonic or compensatory can be difficult by clinical observation alone, and even more confusing in the setting of bilateral lower-limb dystonia. Recent work has used electromyography (EMG) to better characterize dystonic muscles in repetitive-exercise dystonia [11]. In this retrospective case series, we present surface EMG and joint kinematic data from thirteen patients with RD who have undergone instrumented gait analysis (IGA) at the Functional and Biomechanics Laboratory at the National Institutes of Health (NIH). Four cases of RD are described in greater detail to demonstrate the potential utility of EMG with kinematic studies to identify dystonic muscle groups in RD. Lateral heel whip, a proposed novel presentation of lower-limb dystonia, is also described. 

**Figure 1:**
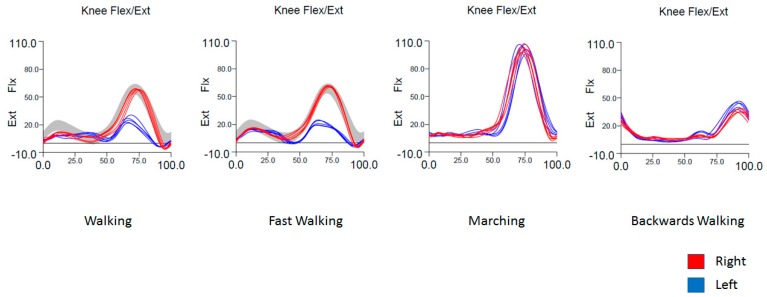
Example of task specificity in a patient with runner’s dystonia. There is impaired flexion of the left knee in swing phase when walking and fast walking when compared to the contralateral leg and to normal values (gray band). Knee flexion is normal when marching/high stepping and when walking backwards, based on symmetry between both sides.

Currently, there are no evidence-based guidelines for assessing focal dystonia with IGA [12]. It is well accepted, though, that dystonia is characterized by prolonged muscle contraction causing sustained movements and abnormal postures [9]. We therefore reasoned that any limb or muscle group suspected of being dystonic should have abnormal posture or movement on kinematics with time-locked EMG abnormalities in muscles that contribute to the abnormal posture or movement. After evaluating each patient clinically, our strategy was to target those muscles with the greatest degree of abnormality on kinematics and EMG. Dystonia was most convincing on kinematics when an abnormality was task-specific, i.e., increased in frequency or amplitude with running, or absent when marching or walking backwards (Figure 1). We graded EMG qualitatively based on previous studies of focal dystonia [11,13], grading onset (inactive, early, late), bursting pattern (phasic, tonic), and duration (unsustained, prolonged, continuous) relative to normative values and compared with the opposite leg. In muscles with abnormal EMG activity, we considered those muscles that inappropriately coactivated with their antagonist muscles the most likely to be dystonic [14,15,16]. Abnormal unilateral leg movement of virtually any etiology, (e.g., spasticity, osteoarthritis), can result in compensatory gait mechanics in the opposite leg [8,17,18], and we therefore expected compensatory movements that were outside the range of normal in other body segments. We assumed that a limb or muscle group was compensating when it was not task-specific (e.g., movement did not increase in amplitude or frequency with running), or if the corresponding EMG pattern was not convincing for dystonia (e.g., lack of inappropriate coactivation with antagonist muscles). We also borrowed from commonly recognized compensatory mechanisms in patients with spastic monoplegia and diplegia to help identify similar compensatory patterns in our patients [19,20]. In cases where the movements were complex and IGA was not definitive, we relied upon our clinical impression of what we thought was most likely to be dystonic. In these situations, the patients’ reports of where they felt tightness or discomfort was often helpful and was always taken into account. Multiple trials with botulinum toxin were required to reach the optimum treatment regimen in all cases. 

## 2. Results

Thirteen patients with RD (six females, seven males) were evaluated in the NIH Functional and Biomechanics laboratory (Table 1). All were avid runners who trained 30–100 miles/week. The mean age of RD onset was 50.5 ± 8.8 years, with a median disease duration of five years at the time of examination. Nine patients had a motor or sensory trick. On evaluation, ten patients had unilateral dystonia, one patient had bilateral dystonia, and two patients had pure truncal dystonia. The most common abnormalities seen on IGA included five patients with excessive knee extension, five with ankle inversion, and four with plantar flexion (Table 2). Nine patients received BoNT therapy based on the results of IGA, and seven out of nine reported some benefit. Three patients reported significant benefit (70–90% improvement), allowing them to continue long-distance running. The remaining four patients reported variable levels of benefit, allowing some to continue training at lower intensity levels while others reported only a short-lived treatment response. Two patients had no response to BoNT at high doses.

### 2.1. Case 1 (RD1): Left Knee Flexion and Bilateral Hip Adduction (Video S1: Case 1)

A 56-year-old nationally-ranked marathon runner presented with a four-year history of involuntary movement in his left leg while running. With what initially presented as an odd sensation of diminished power and lethargy, soon manifested as clumsiness after running short distances. He had difficulty extending the left leg, and the left forefoot tended to strike the right medial ankle. At first the problem was specific to running, but over time it affected walking at normal speed, especially when traversing long corridors. His symptoms transiently improved when he concentrated on tucking his belly, striking the floor with his heel, and curling his toes, which proved difficult to sustain. He continued to run with a considerable drop in performance. He presented to an orthopedic surgeon and later to a neurologist, who noted focal dystonia in the left leg. On initial clinical examination, he was thought to have excessive ankle inversion, causing the left foot to scrape against the right medial ankle. He was injected into the tibialis posterior over multiple sessions up to a dose of 100 units, without any benefit. A trial of carbidopa/levodopa did not relieve symptoms. Trihexyphenidyl helped some, but the side effects were intolerable. 

**Figure 2:**
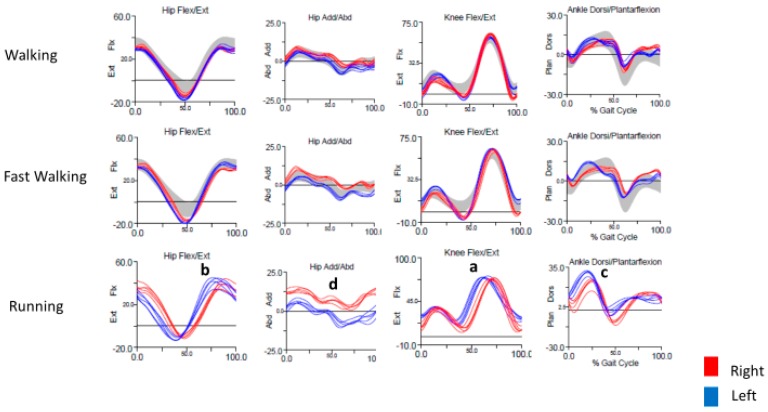
Kinematics for Case 1, showing excessive and early left hip and knee flexion (**a**,**b**), increased left ankle dorsiflexion at midstance, and impaired left plantarflexion at toe-off (**c**). Hip adduction is increased at initial stance and terminal swing, more so on the right (**d**). These abnormalities were not present with walking and were most prominent with running.

**Figure 3:**
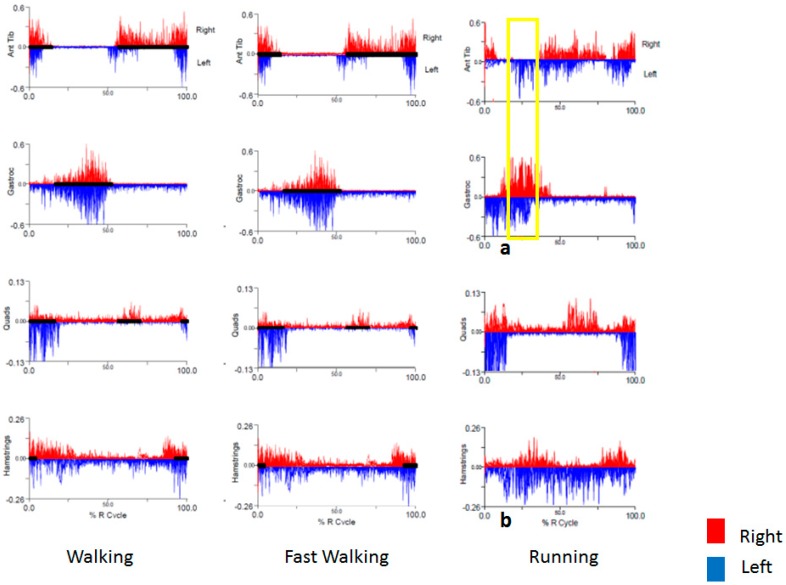
Surface EMG for Case 1, showing continuous activity in the left hamstrings (**b**) and early activity in the left tibialis anterior during running (**a**), along with coactivation of the left gastrocnemius that was not present during the same interval on the right (gold box).

The primary abnormality seen on kinematics was increased left knee flexion during running (Figure 2a) that persisted throughout the gait cycle. This was supported by temporospatial parameters indicating a task-specific reduced mean step length on the left, as well as continuous EMG activity in the left hamstrings (Figure 3b). Left hip flexion also appeared to be shifted earlier in the gait cycle (Figure 2a), but it was unclear whether this was dystonic or compensatory. There was no EMG performed on the hip flexors. What was earlier thought to be ankle inversion as the cause for the left foot to scrape the right ankle turned out instead to be excessive hip adduction. Hip adduction was increased bilaterally, more so on right (Figure 2d) at initial contact and terminal swing, during running only. There was no abnormal inversion of the ankle. While dystonic hip adduction was present, it had less impact on his gait than dystonic left knee flexion. Increased hip adduction was not present with every stride, and mean stance width did not differ between walking and running trials. Interestingly, there was also some increased left ankle dorsiflexion throughout stance, with impaired plantar flexion at toe-off. This was accompanied by early EMG activity in the left tibialis anterior during running, along with coactivation of the left gastrocnemius that was not present during the same interval on the right (Figure 3a, gold box). The strategy for BoNT injections was a minimalist approach, to avoid excessive weakness in this competitive runner. We targeted the left hamstrings, as persistent knee flexion with shortened step length appeared to be the primary dystonic movement that most greatly affected his gait. Increased hip adduction was a secondary dystonic movement that was present bilaterally, and although hip adduction was greater on the right, we targeted the left adductor longus first to limit injections to one leg. BoNT injections into the left semimembranosus, semitendinosus, and adductor longus muscles (Table 3) provided moderate benefit and allowed him to continue running marathons at a slower than previous pace. He seemed to respond well to these injections and has continued on this regimen with sustained benefit. In the event that the current treatment strategy no longer controls proximal hip adduction, causing his feet to scrape against each other frequently or forcefully while running, the right adductor longus would be the next logical muscle to be targeted. Additionally, if excessive ankle dorsiflexion begins to be a problem for him, we would also add the left tibialis anterior muscle, based on the results of the gait study. 

### 2.2. Case 2 (RD10): Right Plantar Flexion and Knee Hyperextension (Video S2: Case 2)

A 72-year-old right-handed male presented with 17 years of running difficulty. Before the onset of symptoms, he was running 70–80 miles per week and competed in 55 marathons over 25 years. His initial symptom was an inability to slow down when running downhill, leading to falls. His symptoms were soon apparent when running on level ground. He felt difficulty lifting the right leg and advancing it forward. He frequently hyperextended the knee with load bearing. Touching the right thigh provided temporary relief. Within a few years, he was no longer able to run, and even walking became difficult. He used walking poles, which seemed to help him move the right leg faster. Fortunately, he successfully switched over to long-distance bicycling. Per his medical records, genetic testing for DYT-1 and DYT-2 was negative. He received three sessions of BoNT prior to coming to NIH. In the first session, he received 150 units into the gastrocnemius and 50 units into the soleus. A month later, he received 25 units into the vastus medialis, 25 units to vastus lateralis, and 50 units to the rectus femoris. Later that year, he received 30 units to the extensor digitorum longus, 30 units into the extensor hallucis longus, and 75 units into the tibialis anterior. He reported no benefit with the injections. In addition to these symptoms, he had evidence of a mild ataxia on balance testing. MRI brain revealed mild atrophy of the cerebellar vermis. There were no other signs of ataxia on examination and he had no relevant family history. He also had signs of a length dependent polyneuropathy. He did report of a prior history of heavy drinking. 

**Figure 4:**
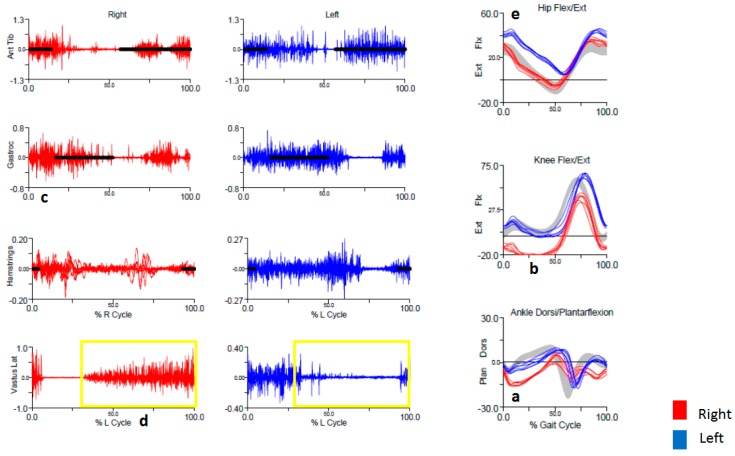
Surface EMG and kinematics for Case 2, showing excessive right plantarflexion (**a**), excessive right knee extension (**b**), early phasic activity in the right gastrocnemius (**c**), and tonic activity in the right vastus lateralis that is essentially not present contralaterally (**d**, gold box). Kinematics also reveal excessive right hip flexion (**e**).

He was referred to the NIH for formal gait evaluation. Kinematics revealed excessive right plantar flexion in early midstance (Figure 4a) and excessive knee extension peaking in late midstance (Figure 4b). There was also less right knee flexion during swing compared to the left. EMG revealed phasic bursts of the right gastrocnemius at initial contact and coactivation with the right tibialis anterior (Figure 4c). The right vastus lateralis had increased tonic activity from midstance through swing phase, which was not present on the left side (Figure 4d, gold box). The left gastrocnemius and tibialis anterior displayed prolonged tonic activation, but since kinematics of the left ankle were normal this was considered compensatory. The primary dystonia was thought to be in the right plantar flexors, with a secondary dystonic movement in the right knee extensors. Knee hyperextension upon load bearing was suggestive of pathologic “plantar flexion-knee extension coupling” as a contributing factor to the hyperextension of his knee, though we still thought that the quadriceps were dystonic based on the gait study. Normally, the quadriceps contract in a compensatory action to prevent buckling of the knee when striking the ground with the heel. In the case of excessive plantar flexion, as seen in spasticity, the forefoot strikes the ground first and bears most of the load, resulting in knee hyperextension when the quadriceps activate [21]. The patient was provided with an ankle-foot orthotic to prevent plantar flexion which did reduce the amount of knee hyperextension. BoNT was tried again, with injections into the gastrocnemius and tibialis posterior, which were thought to be the primary dystonic muscles causing plantar flexion. The vastus lateralis, medialis, and rectus femoris were also targeted with the goal of reducing knee hyperextension (Table 4). He returned to the NIH almost a year later, not recalling a significant benefit from the injections.

On his follow-up visit, he mentioned a curiosity when performing leg raises while lying supine, and showed us in the clinic. At first, his legs were symmetric with normal flexion at the hips and knees, but after a few repetitions, the left hip flexed more than the right, while the right knee went into extension. This was not initially recognized, but on review of his kinematic studies, indeed it revealed excessive left hip flexion in early stance phase (Figure 4e). There was no EMG recording done of the hip flexors at the time of the study. He did not return for BoNT to be attempted in the hip flexors. 

The elusive benefit to therapy in this instance was likely due to a combination of factors. First, this was a challenging case. He had a long-standing history of dystonia that was likely bilateral, (right knee extension, Figure 4b, and left hip flexion, Figure 4e) with multiple compensatory mechanisms that evolved over 17 years. Second, inconsistent follow-up made the fair assessment of treatment effect and the possibility of treatment optimization with BoNT difficult. Had there been an opportunity for further therapy, we would have considered targeting additional muscles, such as the left hip flexors. Lastly, he had signs of mild ataxia detected on balance testing and mild peripheral neuropathy, presumably related to a prior history of heavy drinking, that might have complicated his response to therapy. 

### 2.3. Case 3 (RD3): Truncal Dystonia (Video S3: Case 3—Truncal Dystonia)

**Figure 5:**
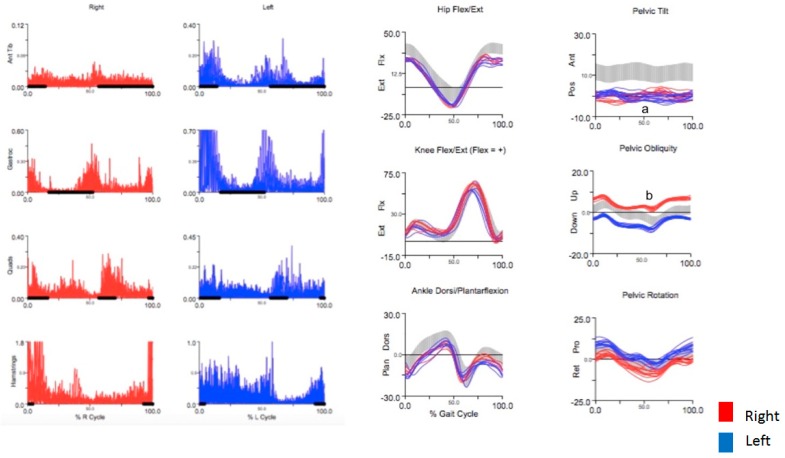
EMG and kinematic studies in a patient with runner’s dystonia presenting as task-specific truncal dystonia. There is abnormal bilateral posterior pelvic tilt (**a**) and upward obliquity of the right pelvis (**b**), consistent with forward and rightward flexion of the abdomen as the primary dystonia. EMG of the lower limbs reveals continuous activity in all muscles with phasic bursts that occur at roughly the same time intervals bilaterally. Kinematics are normal in the lower limbs, supporting a likely compensatory role in the lower limbs.

A 58-year-old man with a 10-year history of long-distance running presented after 1 year of an uncomfortable sensation of stiffness in his groin and hamstrings bilaterally that occurred when running for long distances. The stiffness was associated with flexion at the hip joint. On examination, when walking at a slow pace, there was no obvious abnormality. When walking briskly, he had exaggerated hip and knee flexion with a hard heel strike and excessive dorsiflexion of the ankles and toes. Running appeared even more awkward, but step length was always equal. The initial clinical impression was that he had a focal dystonia in the lower extremities, possibly primarily in the hip flexors. EMG of the lower limbs revealed continuous activity with phasic bursts in all muscles occurring at roughly symmetric time intervals (Figure 5). Kinematics of the lower limbs demonstrated normal movement in the bilateral hips, knees, and ankles. Kinematics of the pelvis revealed posterior pelvic tilt bilaterally (Figure 5a) and upward pelvic obliquity on the right (Figure 5b), representing abnormal forward and rightward flexion of the abdomen. 

Based on the abnormal kinematics of the pelvis, the abdominal muscles controlling pelvic tilt and obliquity were considered the primary dystonic muscles. EMG in the lower limbs was abnormal but symmetric, and since the kinematics of lower limbs were normal, the abnormal EMG pattern in the lower limbs was considered compensatory activity to maintain upright posture during stride. The strategy of BoNT injection was to target predominantly the right abdominal muscles (Table 5). After adequate dose titration, the patient reported significant benefit with a 90% improvement while walking and 70% improvement while running. The functional improvement allowed him to restart running at a slower pace. 

### 2.4. Case 4 (RD9): Left Lateral Heel Whip (Video S4: Case 4—Lateral Heel Whip)

A 50-year-old right-handed woman presented with involuntary posturing of her left leg while running. Her symptoms started at the age of 25, when her mother noticed that she kicked out laterally with her left leg. She had competed in 10K races, marathons, ultra-marathons, 24-h runs, and even a 100-mile run. For years, her “funny stride” was present only while running, but it did not impede her ability to compete. Over the next two decades her pace slowed down and the movements occurred even while walking. She had to concentrate very hard to walk normally and maintain balance. The movement would occur after walking or running for about 10 feet. She had no problems riding a bicycle or swimming and had only mild difficulty using an elliptical machine. She tried a short trial of carbidopa/levodopa without benefit. BoNT was also tried with injections into the “left calf” (muscles not specified in the records), causing weakness but no benefit.

**Figure 6:**
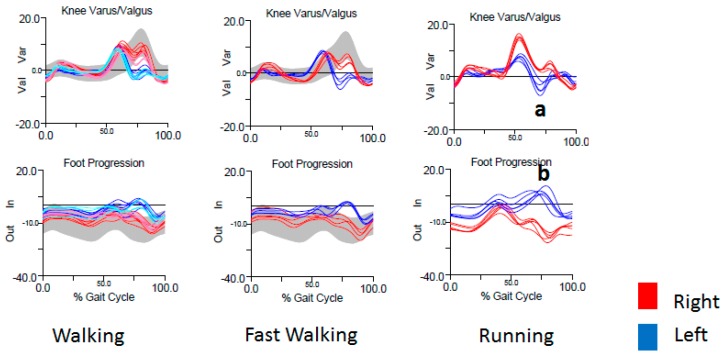
Joint kinematics in a patient with runner’s dystonia presenting as task-specific lateral heel whip. There is increased left knee valgus (**a**) and inward foot progression (i.e., outward heel progression) (**b**) during swing phase. The lateral heel whip is maximal during running.

**Figure 7:**
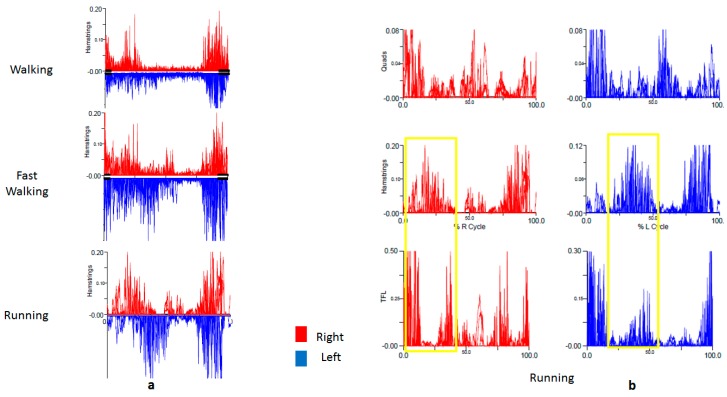
EMG of a patient with runner’s dystonia presenting as task-specific lateral heel whip. There is delayed firing of the left hamstrings that is maximal during running (**a**) along with coactivation of the left hamstrings and TFL (**b**) that is not present on the right (gold box).

She was referred to the NIH for formal gait evaluation. Clinically, she had an outward rotational movement of the left lower leg, causing the heel to whip out laterally when the knee was flexed in swing phase. The movement occurred intermittently with prolonged walking, more consistently with running. It was not present at all when she walked or ran backwards (see Video S4—Case 4 Lateral Heel Whip). Kinematics captured an increased knee valgus just before mid-swing (Figure 6a), followed immediately by increased inward foot progression, (i.e., toe in, heel out) (Figure 6b). Surface EMG demonstrated that when running, the firing time of the left hamstrings was delayed to midstance phase, just before toe-off (Figure 7a), followed by a slight delay in firing of the left quadriceps. There was also coactivation of the hamstrings with the tensor fascia lata (TFL) on the left, which was not present on the right. (Figure 7, gold box).

Interpretation of the IGA in this case is complex. Increased knee valgus in swing phase, followed by immediate inward foot progression, reflects the observed whipping motion of the heel. EMG revealed abnormalities in the upper leg—delayed firing in the hamstrings and coactivation of the hamstrings with the TFL, followed by delayed firing of the quadriceps. However, kinematics of the primary function of these muscles, namely hip rotation (TFL), knee flexion (hamstrings), and extension (quadriceps) were normal. We think that the EMG findings likely represent the secondary functions of these muscles. The TFL flexes the hip and knee during running, while the biceps femoris flexes the knee and externally rotates the tibia when the knee is flexed. Coactivation of the TFL and biceps femoris could result in a dystonic posturing of the lower leg (increased valgus during mid swing). This is followed by a delayed firing of the quadriceps, which in addition to extending the knee, causes inward progression of the foot with a whipping motion, as is seen normally when kicking. 

Heel whip is defined by a medial or lateral rotation of the foot in the transverse plane during swing phase and is named by the direction of movement of the heel [22]. Lateral heel whip can be a compensatory movement allowing for rapid knee extension and was originally described in patients with below-the-knee amputations and prosthetic lower limbs [7]. Lateral and medial heel whip is also seen in recreational runners, presumably due to weak and underdeveloped stabilizing muscles or previous injury [22]. We had a similar patient previously (not included in our series) with a lateral heel whip without EMG correlation, for whom we could not conclusively determine that dystonia was the etiology. In the current patient’s case (RD9), lateral heel whip was thought to be the presenting phenomenology of her dystonia. Unfortunately, she did not follow up after her initial evaluation. If she had, we would have recommended BoNT injection into the left biceps femoris and/or TFL.

## 3. Discussion

IGA serves as an extension of the physical examination. In some cases, a dystonic movement is obvious and the IGA confirms the clinical impression. In more difficult cases, it may help to precisely identify the muscles involved or to distinguish dystonic activity from compensatory activity. For example, in Case 1, the initial clinical impression was that of dystonic ankle inversion in a patient whose left foot repeatedly scraped his right medial ankle mid stride. IGA did not show ankle inversion, but rather it revealed increased hip adduction, and BoNT injections targeted to the adductor longus muscle provided relief while previous injections to the tibialis posterior were ineffective. In Case 3, the detection of severely increased posterior pelvic tilt and right upward pelvic obliquity led to the correct targeting of abdominal muscles with BoNT in a patient with pure truncal dystonia, where on initial clinical evaluation the dystonia was thought to be in the lower extremities. 

The patient in Case 4 presented with a lateral heel whip, a rotational sling-like movement of the tibia when the knee is flexed, which we propose as a novel phenomenology in lower-limb dystonia. Lateral heel whip has been described in other conditions, such as in below-the-knee amputees with prosthetic limbs and in runners with proximal hip weakness or injury. In this case, however, our patient (RD9) displayed the movement in a task-specific manner (see video) that was supported by EMG and kinematic analysis, leading us to conclude that it was most consistent with dystonia.

In this series, we attempted to describe the EMG and joint kinematic patterns in patients with a clinical history most consistent with runner’s dystonia, characterized by multiple years of long-distance running preceding the onset of a task-specific focal or lower segmental dystonia, often with the presence of geste antagoniste and an absence of other organic findings. None of the patients in our series had a relevant family history or presence of other neurologic findings that would lead us toward a diagnosis other than RD. Other conditions that may present with focal lower extremity dystonia should be considered, for example, paroxysmal exercise induced dystonia (PED) [23], paroxysmal kinesogenic dyskinesia (PKD) [24], Parkinson disease (PD) (especially early onset PD) [23,25], dopa-reponsive dystonia (DRD) [26], and GLUT-1 deficiency [27,28]. Generally, a family history and early age of onset suggests a diagnosis other than RD. Responsiveness to other therapies is also another clue. PD and DRD respond to Levodopa, PKD may respond to anticonvulsants, and GLUT-1 may respond to a ketogenic diet. The latency and duration of the dystonia relative to exercise may differ from RD. PKD is often triggered by sudden movements or startle and is usually of short duration, while PED may present with a more prolonged duration, often persisting for some time after the exercise has stopped [29]. Task-specificity is not a feature of PKD, PED, or GLUT-1 deficiency but may be present in generalized dystonias. The dystonia may have diurnal fluctuation, as can be seen in DRD or PD. The presence of other clinical features, such as bradykinesia, tremor, and rigidity would suggest PD, while other movement disorders triggered by movement (chorea, athetosis, ballism) may suggest PKD [29]. The presence of dystonia in the upper limbs would suggest a generalized dystonia, and the presence of epilepsy or encephalopathy may suggest GLUT-1 deficiency [27]. Genetic testing may be helpful in familial forms of PKD, PED, and PD and is diagnostic in DRD and GLUT-1. A low CSF glucose is also seen in GLUT-1 deficiency [27,28]. DATscan can be helpful to identify PD [23], especially early in the disease course when the cardinal features are less prominent. 

This study had some limitations. First, this is a retrospective case series and treatment effects are limited to clinical description alone. We did not systemically test outcomes with post-injection IGA, and there were no controls for comparison. Follow up was also limited in some cases. Only 9 out of 13 received BoNT based on the results of IGA. Second, there are no evidence-based guidelines for identifying dystonia with IGA, but our approach seems reasonable based on what is currently known about dystonia. We considered a limb or muscle group as dystonic when it had abnormal kinematics, with at least some degree of task specificity, that corresponded well with abnormal EMG activity and coactivation in antagonist muscles. In some cases, temporospatial parameters, (e.g., step length/time, step width, stance/swing time) provided supportive evidence for identifying the most dystonic muscles—the muscles with the greatest impact on the patient’s gait. Abnormalities that were not present in both kinematics and EMG, especially if they lacked task specificity or coactivation, were more likely to be considered compensatory. Future prospective studies systematically testing the utility of IGA in lower-extremity dystonia using this approach should be done. 

BoNT is the mainstay of treatment for task-specific dystonia [30] and is best offered as one part of a comprehensive treatment plan, including the use of orthotics, walking or running aides, and physical rehabilitation. All of the patients in our case series were evaluated by physical therapists and were given individualized treatment plans. In difficult cases, in which there is no apparent improvement of running ability with treatment, patients who wish to continue an exercise regimen may find satisfaction by switching to other sports, such as swimming or cycling, as was the case for some of the patients in this series. Similar to reports of other focal task-specific dystonias [31], the treatment response to BoNT in RD is often disappointing [11]. Yet, of the nine patients who received BoNT therapy guided by IGA, seven reported some benefit and three patients reported significant benefit (70–90% improvement), allowing them to continue long-distance running. While not tested systematically, the results in this case series suggest that IGA may have a role to play in the diagnostic and treatment plan in patients with task-specific lower extremity segmental dystonia. 

## 4. Materials and Methods

**Figure 8:**
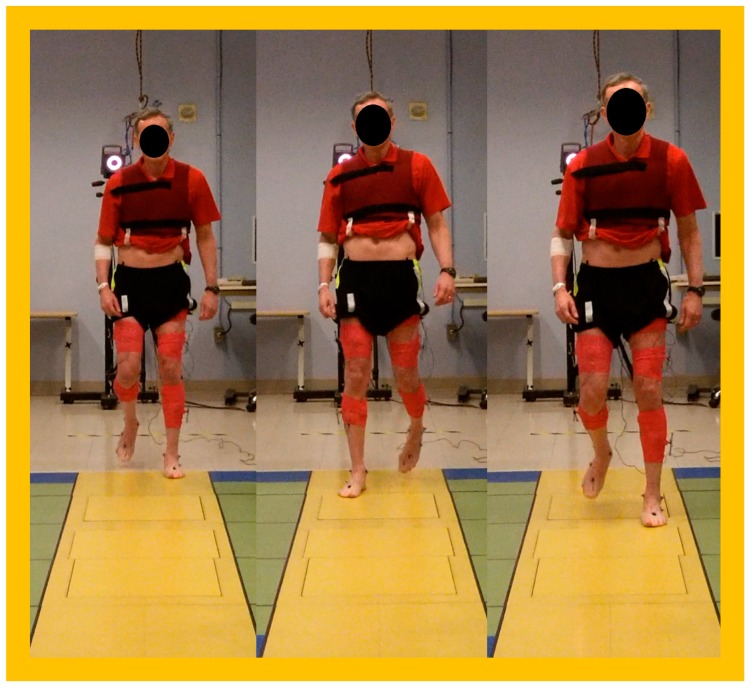
Still frames showing a patient with runner’s dystonia (RD13) at the Biomechanics Laboratory at the National Institutes of Health (NIH).

For kinematic analysis, reflective markers were placed on the lower-limb segments or joints using a standardized marker set [32] (Figure 8). Three-dimensional marker position was collected in real time with an array of 10 Vicon infrared cameras. C-Motion Visual3D software was used to process motion capture data. Joint angles were calculated for the pelvis, hips, knees, and ankles and were compared to laboratory normative data collected from a large group of healthy volunteers, represented as a gray band (Figure 1) overlaid on patient tracings. Quantitative analysis of gait parameters, such as step length and width, swing and stance time, double limb support time, and gait speed was also performed. Real-time video recording was obtained where possible. 

Using a wireless EMG system, surface electrodes were placed over four standard leg muscle groups bilaterally (tibialis anterior, gastrocnemius, quadriceps, and hamstrings). Additional muscles were sampled depending on the patient’s clinical presentation, and in some cases, fine wire EMG placed under ultrasound guidance was used for deeper muscles lacking a surface representation. Dynamic EMG over multiple strides was averaged and normalized to one gait cycle. Periods of normal firing time based on pooled normative data from healthy controls in the lab database were represented as black solid lines and were superimposed over each subject’s tracing for visual comparison.

All subjects gave their informed consent for inclusion before they participated in the study. All participants who were video recorded or photographed provided signed written authorization beforehand. The study was conducted in accordance with the Declaration of Helsinki and the protocol was approved by the National Institute of Neurological Disorders and Stroke (NINDS) Institutional Review Board.

## Figures and Tables

**Table 1:** Characteristics of RD patients.

RD	M/F	Age at Onset	Disease Duration	Sensory/Motor Trick	Prior Injury	Average Distance (Miles/Week)	Most Effective Treatment	Average Dose of botulinum neurotoxin (BoNT) *
1	M	52	10	Motor	No	50–70	BoNT	246
2	M	54	9	Motor, Sensory	No	100	BoNT, weighted backpack	407
3	M	57	5	Sensory	Yes	60	BoNT, clonazepam	293
4	M	53	2	Motor	No	50	BoNT	227
5	F	58	2	None	No	30	None effective (Levodopa tried)	Unknown
6	F	43	3	Motor	No	Unknown	BoNT, physical therapy	122
7	F	51	5	None	No	55	None tried	Not tried
8	F	46	1	Motor	No	27.5	BoNT	120
9	F	25	25	None	No	32.5	None effective (BoNT tried once, Levodopa tried)	Unknown
10	M	55	17	Sensory	No	75	Ankle foot orthotic	425
11	F	50	5	Sensory	No	Unknown	BoNT	50
12	M	56	8	None	No	37.5	None effective (Levodopa tried)	Not tried
13	M	56	3	Motor, Sensory	Yes	60	None effective (Levodopa and Carbamazepine tried)	515

* Onabotulinum-A, 100 units/1 mL normal saline.

**Table 2:** Clinical, Kinematic, and Electromyography (EMG) characteristics of RD patients.

RD	Clinical Presentation	Kinematics	EMG
1	Left forefoot striking the right medial ankle, decreased running speed	Increased right hip adduction at initial stance and terminal swing, excessive and early left hip and knee flexion, increased left ankle dorsiflexion at midstance, impaired left plantarflexion at toe-off.	Continuous activity in left hamstrings, early phasic activity in left tibialis and coactivation with left gastrocnemius. (Hip adductors not recorded)
2	Trunk flexion, rightward rotation and tilt	Posterior pelvic tilt, phasic trunk flexion, increased right upward pelvic obliquity, rightward pelvic rotation, decreased bilateral hip flexion.	Phasic bursts of right rectus abdominus, time-locked with phasic trunk flexion seen on kinematics.
3	Severe forward and rightward flexion of the abdomen.	Posterior pelvic tilt, increased upward obliquity of right pelvis.	Continuous activity with phasic bursts occurring at roughly symmetric time intervals in bilateral tibialis anterior, gastrocnemius, quadriceps and hamstrings.
4	“Trotting gait” leading with the left leg.	Right external hip rotation, increased right knee flexion in stance and left knee flexion during swing, bilateral external knee rotation, increased right ankle dorsiflexion in stance.	Phasic bursts in right hamstrings during late stance and in the left hamstrings during midstance to early swing. Increased activity in right tibialis anterior in stance.
5	Stiffness and inability to flex left knee.	Decreased left knee flexion and plantar flexion in late-stance phase (toe-off). Present only with walking and brisk walking, (unable to run), corrects with marching and walking backwards.	Burst in left quadriceps in late-stance phase, coactivation with left hamstrings. Early onset activity in left tibialis anterior in swing phase.
6	Left plantar flexion and ankle inversion.	Increased left plantar flexion and inward foot progression during swing phase.	Prolonged activity of left gastrocnemius from midstance phase to mid swing phase. Coactivation with tibialis anterior.
7	Stiffness, limited right knee flexion.	Decreased right flexion during swing phase.	Burst activity in right gastrocnemius and coactivation with hamstrings in mid swing phase.
8	Left knee extension, plantar flexion and ankle inversion.	Plantar flexion in early-stance phase (heel strike). Swing time increased in trials when attempting to compensate for plantarflexion.	Early onset activity in left gastrocnemius, tibialis posterior and flexor digitorum longus on early stance phase (heel strike).
9	Left lateral heel whip.	Increased knee valgus in swing phase, followed by immediate inward foot progression.	Delayed firing of left hamstrings with activation of the tensor fascia lata in late stance before toe-off, followed by delay in firing of left quadriceps.
10	Right foot dragging, inability to stop when running downhill, difficulty planting right heel.	Increased right hip flexion, knee extension in late midstance, plantar flexion in early midstance.	Early phasic activity in right gastrocnemius, increased tonic activity in the right vastus lateralis.
11	Left plantar flexion and ankle inversion, left toe curls in and clips right ankle.	Left ankle inversion during mid-swing phase.	Burst activity in left flexor digitorum longus in early swing phase. Coactivation with tibialis anterior.
12	Stiffness and toe dragging bilaterally. Bilateral ankle inversion.	Increased bilateral ankle inversion throughout gait cycle, (left greater than right), decreased knee flexion in early stance.	Increased phasic activity in bilateral quadriceps (Left greater than right), in stance phase. Ankle invertors not recorded.
13	Left knee extension, plantar flexion and ankle inversion.	Increased knee extension in late stance, increased plantar flexion and inward foot progression in mid swing.	Burst activity in left rectus femoris in midstance (absent on right). Increased activity of left gastrocnemius and coactivation with tibialis anterior in mid to late swing.

**Table 3:** BoNT muscle selection for Case 1 (RD1). Total 200 units, onabotulinum A.

Muscle	Side	Dose per Muscle (Units)	Number of Sites per Muscle
Adductor longus	Left	30	1
Semimembranosus	Left	85	2
Semitendinosus	Left	85	2

**Table 4:** BoNT muscle selection for Case 2 (RD10). Total 425 units, onabotulinum A.

Muscle	Side	Dose per Muscle (Units)	Number of Sites per Muscle
Medial gastrocnemius	Right	150	3
Lateral gastrocnemius	Right	75	2
Tibialis posterior	Right	50	1
Rectus femoris	Right	50	2
Vastus lateralis	Right	50	1
Vastus medialis	Right	50	1

**Table 5:** BoNT schedule for Case 3 (RD3). Total 300 units, onabotulinum A.

Muscle	Side	Dose per Muscle (Units) 2:1 Dilution	Number of Sites per Muscle
Rectus Abdominus	Right	200	8
Rectus Abdominus	Left	40	1
External Abdominal Oblique	Right	25	1
External Abdominal Oblique	Left	10	1
Internal Abdominal Oblique	Right	25	1

## Supplementary Materials

The following are available online at http://www.mdpi.com/2072-6651/10/4/166/s1, Video S1: Case 1; Video S2: Case 2; Video S3: Case 3—Truncal Dystonia; and Video S4: Case 4—Lateral Heel Whip.
